# Forecasts for the concentration of petroleum gas leakage diffusion under different liquid level heights of a sealing ring of sizeable floating roof tank

**DOI:** 10.1038/s41598-022-22934-2

**Published:** 2022-11-09

**Authors:** Yonghui Wei, Wenfeng Wu, Hongliang Yu, Jinshu Lu, Mengqing Huang, Min Guo

**Affiliations:** 1 Department of Naval Architecture and Maritime, Zhejiang Ocean University, Zhoushan, 316022 China; 2 Department of Mechanical and Aerospace Engineering, Brunel University, London, England; 3 School of the Ocean, Yantai University, Yantai, 264005 China

**Keywords:** Environmental impact, Chemical engineering, Environmental sciences, Environmental chemistry, Atmospheric chemistry, Environmental monitoring

## Abstract

The sealing ring of the external floating roof tank is prone to petroleum gas leakage due to material aging and oil corrosion. Petroleum gas leakage and diffusion easily accumulate above the floating deck. When it is within the explosion limit range, there will be the risk of explosion and fire. To deal with the explosion accident of storage tank caused by the concentration distribution of petroleum gas leakage for the sealing ring, and to study the influence of petroleum gas diffusion and concentration distribution after sealing ring leakage on the control area above the floating deck in the tank farm environment, this paper established numerical models of sealing ring leakage under different liquid level heights for 10 × 10^4^ m^3^ external floating roof tank. Through numerical calculation, it is found that the diffusion concentration of petroleum gas is related to the wind speed, the range of the control area above the floating deck, and leakage when sealing rings leak at different liquid levels. Through dimensionless analysis, the functional relationship of gas leakage diffusion concentration distribution under different liquid level heights of external floating roof tank sealing rings is verified by numerical calculation results. The results show that the numerical results are consistent with those predicted by the formula.

## Introduction

According to statistics, our country has built nine National Petroleum Reserve Bases in Zhoushan and Zhenhai (“China has built nine national oil reserve bases to reserve 37.73 million tons of crude oil”^1^). External floating roof tanks for floating decks close to the oil level can significantly reduce evaporation loss in storage. Therefore, it is a national reserve in the extensive use of a type of tank^2^, (Qu et al. 2017). There is a 10-30 cm gap between the floating deck and the tank wall of the external floating roof tank, that is, the sealing ring. Due to disrepair, the sealing ring will corrode and perforate, resulting in the leakage of petroleum gas into the tank. Petroleum gas is a typical volatile organic compound, and its leakage into the atmosphere will cause safety and environmental problems^3–7^. In recent years, there have been many fire and explosion accidents at the seal of the floating deck in China^8,9^, which has seriously affected the safe production of tank farms. According to statistics, most oil tanks with fire accidents are large floating roof tanks about 10 × 10^4^ m^3^ and 15 × 10^4^ m^3^^10^, and the safe operation of large floating roof tanks has become a hot topic of national and social concern. To correctly predict the distribution law of petroleum gas, the risk, and the scope of fire caused by an explosion so that it can provide theoretical support for the prevention and rescue of fire accidents in external floating roof oil storage tanks. Therefore, it is necessary to study the diffusion law of petroleum gas in the space above the sealing ring of a large external floating roof oil storage tank.

Considering the advantages of numerical simulation, such as convenient calculation, scholars at home and abroad have conducted extensive research on petroleum gas leakage and diffusion by using CFD numerical simulation technology. It focused primarily on the characteristics of the petroleum gas diffusion flow field and the fitting of the diffusion concentration function after petroleum gas leakage. In terms of the features of petroleum gas diffusion flow fields, Kountouriotis et al.^11^ discussed the influence of wind speed and other factors on the aspects of diffusion flow fields after petroleum gas leakage in gas stations. Qu et al.^12^ found the petroleum gas leakage and diffusion of the spherical tank area of liquefied petroleum gas and obtained the wind speed distribution and pollutant migration under the effect of airflow. Zhao et al.^13,14^ considered that the wind speed and the height-diameter ratio of the space above the floating plate have a great influence on the wind pressure distribution and wind speed migration law on the floating deck of the external floating roof tank. The wind disturbance law of the space above the floating deck is obtained by simulation when the height-to-diameter ratio is the high, medium, and low liquid level of the tank. Hao et al.^15^ analyzed the petroleum gas leakage and diffusion of the sealing ring of the external floating roof tank and obtained the wind speed migration rule under the action of airflow and the gas diffusion along the tank wall to the space above the floating deck, and the diffusion concentration was as follows: the concentration of both sides of the floating deck was higher than that of the upwind side and downwind side. Zhou et al.^16^ simulated the diffusion concentration after gas leakage in the tank farm and obtained the influence law of environmental wind speed on the gas leakage jet. There is a positive correlation between wind speed and explosion risk area caused by gas leakage. Xiao et al.^17^ used the improved Gauss smoke cluster model to simulate the gas leakage and diffusion process of storage tanks and determined the distribution law of gas concentration in the tank area under different environmental wind speeds and atmospheric stability. According to the dangerous degree of gas concentration, the tank area was divided into the explosion danger area, flash fire danger area, and suffocation danger area. The improved Gaussian smoke cluster model can more accurately reflect the influence of tank group’s environmental conditions on gas concentration distribution and dangerous area rings and can guide the safe operation and management of storage tanks. These studies reveal the influence law of single variable on gas concentration diffusion and migration from the aspects of tank farm environmental conditions and tank factors, which is helpful to understand the distribution law of gas diffusion concentration in tank farm and provide reference for safe operation of tank and further research on gas diffusion law. However, the distribution of gas diffusion concentration in the actual tank farm is limited by the joint action of various influencing factors in complex environmental conditions. At present, the law of gas diffusion influenced by various factors remains to be studied. At the same time, in the fitting of the diffusion concentration function about petroleum gas leakage, Yu et al.^18^ simulated and analyzed the influence of petroleum gas escape velocity on the distribution of petroleum gas diffusion concentration under different pressure conditions in the vault tank, and deduced the functional relationship between petroleum gas concentration and petroleum gas escape velocity. Chen et al.^19^ established a dynamic mathematical diffusion model of heavy gas leakage in storage tanks by using the Gaussian plume model and obtained the dynamic analysis process of gas spatial concentration field at different times. Wu et al.^20^ combined a geographic information system with a Gaussian plume model and established the relationship of concentration distribution function according to the pollutant concentration parameters of each location. Hui et al.^21^ for cylindrical liquefied gas storage tanks, aiming at the root causes of the fire that leads to accidents, such as raising the pressure in the tank, promoting the leakage and aggravating the leakage, based on the mass and energy conservation equation and the pressure vessel rupture prediction formula, established a tank leakage fire failure prediction model verified by actual accidents. This method can judge the accident situation according to the fire type and other factors, and always guard against the explosion caused by tank overpressure failure. Li et al.^22^, aiming at the safety problems caused by the leakage and diffusion of crude oil storage tanks, simulated the relationship between the leakage aperture and the height above the ground, the maximum combustion speed, the flame height and the diameter of the tank, respectively, during the continuous combustion of the tank, and made multivariate nonlinear fitting for the simulation results. The simulation results consider the mutual influence of gas diffusion conditions after crude oil leakage, and provide practical reference for the revision of fire protection code for storage tank design. In the literature about the concentration prediction formula of gas leakage and diffusion, most of the researches are based on the theoretical model to change a single variable to obtain the spatial and temporal distribution of gas concentration field, and the fitting function only aims at the estimation of the accident consequences caused by gas leakage, while ignoring that the concentration prediction formula should be generally applicable to the influence of many factors on gas concentration diffusion under real environmental conditions, as well as the concentration value after gas diffusion and the prediction of rescue time in the whole process of the accident. In addition, Jing et al.^23^ concluded through physical and numerical experiments that when the critical Reynolds number of the wind tunnel test model is equal to the numerical simulation prototype, and The distribution of gas concentration in the wind tunnel experiment and numerical simulation is similar under different liquid level height. It is proved that the experimental values of petroleum gas concentration fields at different liquid level heights can verify the accuracy of the numerical simulation.

In summary, scholars at home and abroad have done a lot of research on the diffusion of petroleum gas leakage in storage tanks from wind speed, tank diameter ratio, Reynolds number, etc. However, the current research mainly focuses on the phenomenon of gas leakage and diffusion in storage tanks under the condition of single variable, without considering the law of petroleum gas concentration diffusion under the combined action of various factors in the tank farm. The distribution function of petroleum gas diffusion concentration in the floating roof tank is only limited to the prediction of gas diffusion concentration under a certain condition, and the prediction of accident consequences (such as the impact caused by fire) after gas leakage, and the prediction of gas leakage diffusion concentration before the accident has not been considered. In this paper, the effects of Reynolds number (Re), wind speed, liquid level height, and leakage on the diffusion concentration of petroleum gas leakage are analyzed numerically when petroleum gas leakage occurs in the sealing ring of the external floating roof tank at different liquid levels. Combined with the influencing factors in the Gaussian theory formula, the influencing parameters are quantified, and an effective distribution function formula of petroleum gas concentration is derived. This method is suitable for predicting the concentration distribution of petroleum gas leakage and diffusion in storage tanks with different layouts and volumes. This method can provide a reference for the environmental safety assessment of oil storage tank areas.

## Model establishment and numerical method

### Mathematical model

Crude oil and its products are mixtures of various compounds. The volatile petroleum gas components will change with the change in external conditions such as temperature and oil properties. Therefore, it is a highly complex process of quantitative description and numerical solution to simulate the actual leakage of petroleum gas from a single floating deck seal to an external floating roof tank. To solve the mathematical model, this paper makes the following assumptions:

- The wind speed in the external environment does not change with time;
- Ignore the influence of ground roughness on petroleum gas diffusion;
- Ignore the resistance of petroleum gas leakage through the sealing ring.

Oil–gas leakage diffusion is the process of volatile organic compounds diffusion (VOCs), and the main components of VOCs are C3–C5. As the main component of oil–gas diffusion, propane has similar physical properties (density, viscosity, diffusion coefficient, etc.) to VOCs. In order to simplify the calculation, propane is used as a single component in the process of oil–gas diffusion for simulation calculation.

The diffusion of petroleum gas components is described by a single-phase, multi-component transport model. Therefore, the main governing equations of this flow include continuity equation, momentum conservation equation, energy conservation equation, component transport equation, and turbulence equation^24^.

1. Continuity equation
  1 $$\frac{\partial \rho }{\partial t}+\frac{\partial }{\partial {x}_{j}}(\rho {u}_{j})=0$$
  where ρ is the density of mixed gas, kg/m^3^; ${x}_{j}$ is the movement corresponding to x, y, and z directions respectively; ${ u}_{j}$ is the velocity component in x, y, and z directions, respectively, m/s.

(2) Momentum conservation equation
  2 $$\frac{\partial (\rho {u}_{j})}{\partial t}+\frac{\partial }{\partial {x}_{j}}\left(\rho {{u}_{i}u}_{j}\right)=-\frac{\partial p}{\partial {x}_{j}}+\frac{\partial }{\partial {x}_{j}}\left({\mu }_{t}\frac{\partial {u}_{i}}{\partial {x}_{j}}\right)+(\rho -{\rho }_{a}){\mathrm{g}}_{i}$$
  Type, p is the absolute pressure, pa; μt is the dynamic viscosity of the fluid, Pa·s; ρ_a_ is the air density, kg/m^3^; g_i_ is the acceleration component of gravity in x, y, and z directions, m/s^2^.

(3) Energy conservation equation
  3 $$\frac{\partial (\rho T)}{\partial t}+\frac{\partial }{\partial {x}_{j}}(\rho {u}_{j}T)=\frac{\partial }{\partial {x}_{j}}\left(\frac{{\mu }_{t}}{{\sigma }_{c}}\frac{\partial T}{\partial {x}_{j}}\right)+\frac{{C}_{{P}_{v}}-{C}_{{P}_{a}}}{{C}_{P}}\left[\left(\frac{{\mu }_{t}}{{\sigma }_{c}}\right)\frac{\partial \omega }{\partial {x}_{j}}\right]\frac{\partial T}{\partial {x}_{j}}$$
  where T is the temperature of the fluid, K; σ_c_ is the Schmidt number of turbulent flow, usually 1.0; σ_T_ is the rough Prander number, usually 0.9 ~ 1.0; Cp, Cp_a,_ and Cp_v_ are the constant pressure-specific heat capacity of mixed gas, continuous pressure specific heat capacity of air and constant pressure specific heat capacity of leaked petroleum gas respectively, J/(kg·K); ω is the mass fraction.

(4) Component transport equation
  4 $$\frac{\partial (\rho \omega )}{\partial t}+\frac{\partial }{\partial {x}_{j}}\left(\rho {u}_{j}\omega \right)=\frac{\partial }{\partial {x}_{j}}(\rho {D}_{1}\frac{\partial \omega }{\partial {x}_{j}})$$
  where Dl is the turbulent diffusion coefficient, m^2^/s.

(5) Turbulence model
  5-1 $$\frac{\partial }{\partial t}\left(\rho k\right)+\frac{\partial }{\partial {x}_{j}}\left(\rho {ku}_{j}\right)=\frac{\partial }{\partial {x}_{j}}\left[\left(\mu +\frac{{\mu }_{t}}{{\sigma }_{k}}\right)\frac{\partial k}{\partial {x}_{j}}\right]+{G}_{k}+{G}_{b}-\rho \varepsilon -{\gamma }_{M}+{S}_{k}$$
  5-2 $$\frac{\partial }{\partial t}\left(\rho \varepsilon \right)+\frac{\partial }{\partial {x}_{j}}\left(\rho \varepsilon {u}_{j}\right)=\frac{\partial }{\partial {x}_{j}}\left[\left(\mu +\frac{{\mu }_{t}}{{\sigma }_{\varepsilon }}\right)\frac{\partial \varepsilon }{\partial {x}_{j}}\right]+{C}_{1\varepsilon }\frac{\varepsilon }{k}\left({G}_{k}+{C}_{3\varepsilon }{G}_{b}\right)-{C}_{2\varepsilon }\rho \frac{{\varepsilon }^{2}}{k}+{S}_{\varepsilon }$$
  where k is turbulent kinetic energy, m^2^/s^2^; ε is the dissipation rate; G_k_ is the generic term of rough kinetic energy k caused by average velocity gradient; Gb is the generic term of rough kinetic energy k caused by buoyancy; γ_M_ represents the contribution of pulsating expansion incompressible turbulence; C_1ε_, C_2ε_, and C_3ε_ are empirical constants; σ_k_ and σ_ε_ are the Prander numbers corresponding to turbulent kinetic energy k and dissipation rate ε respectively; S_k_, S_ε_ user-defined.

### The theoretical model of gas diffusion

Common gas diffusion models include the Gaussian model^25^, box model^26^, shallow model^27^, and CFD model^28^. Among them, the Gaussian plume model has the characteristics of a small amount of calculation, fast simulation speed, accurate simulation data, and the ability to calculate the gas diffusion for a while. This paper takes it as the theoretical model of gas diffusion, and the model formula is as follows:

(6) Gas diffusion model
  6 $$C\left(x, y,z, H\right)=\frac{Q}{2\pi u{\sigma }_{y}{\sigma }_{z}}exp\left(-\frac{{y}^{2}}{2{\sigma }_{y}^{2}}\right)\left\{exp\left(-\frac{{\left(z-H\right)}^{2}}{2{\sigma }_{Z}^{2}}\right)\right.+exp\left(\frac{{(z+H)}^{2}}{2{\sigma }_{Z}^{2}}\right)$$
  where H is the influential source height, u is the average wind speed at the source height, and σ_y_ and σ_z_ are the lateral diffusion parameters and vertical diffusion parameters, respectively. Q is the source intensity (for continuous source emission, it refers to the emission per unit time).

### Physical model and boundary conditions

#### Establishment of a physical model and setting of boundary conditions

**Figure 1:**
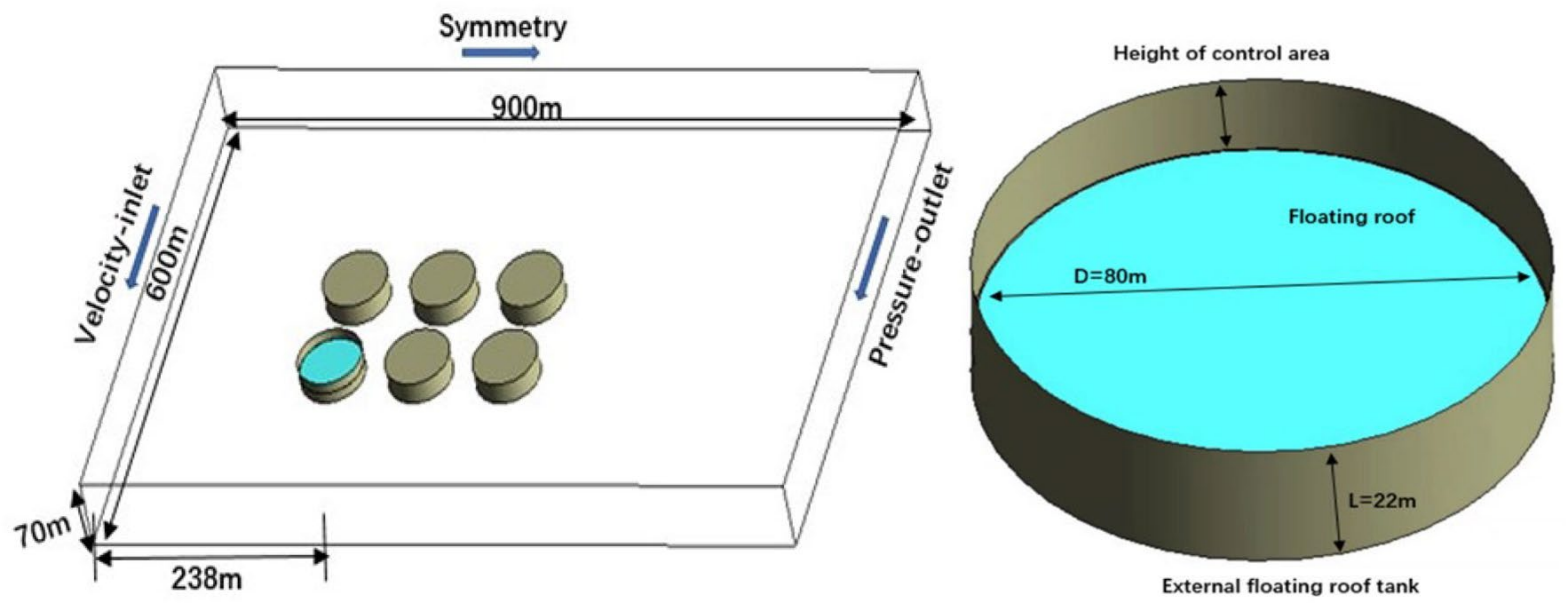
Physical model.

Because it is difficult to completely copy the details of the tank groups site when modeling, the model is simplified according to the actual situation. Gambit software is used to build the 3D model of the external floating roof tank at about 10 × 104m3 scale according to the ratio of 1:1, as shown in Fig. 1. The calculated domain size is 900 m × 600 m × 70 m, and the tank diameter D is 80 m and the tank height L is 22 m. The tank distance is 32 m (the tank distance is set according to the Standard for Fire Protection Design of Petrochemical Enterprises^29^; the Code for Design of Oil Depot (Sinopec Group 2014)^30^ and the width of the sealing ring is 30 cm. The external wind direction is along the positive direction of the x-axis. The wind blows in a specific direction to the external floating roof tank, causing the pressure difference to change in the upper space of the floating deck and around the tank wall, which belongs to turbulent flow and is described by the k-ε turbulence model. Tetrahedral meshes are used to divide the model. The parameters of boundary conditions and initial conditions are set, as shown in Table 1.

**Table 1:** The setting of boundary conditions and initial conditions.

Boundary conditions	Setting boundaries	Initial condition	Parameter setting
Calculation domain left entrance (right exit)	Velocity-inlet (pressure-outlet)	Temperature	300 K
Calculate the top and both sides of the domain boundary	Symmetry	Atmospheric pressure	101325 Pa
		Atmospheric stability	D(− 1 °C/m)
Tank wall and ground	Wall	Mass flow of leakage port	10 m/s
Tank top	Interior	Wind speed [satisfy the formula^25^]	${u}_{z}={u}_{1}\frac{lnz-ln{z}_{0}}{ln{z}_{1}-ln{z}_{0}}$
Sealing ring	Velocity-inlet		

Model and algorithm: k-ε model, Simple standard algorithm.

#### Grid independence verification

**Figure 2:**
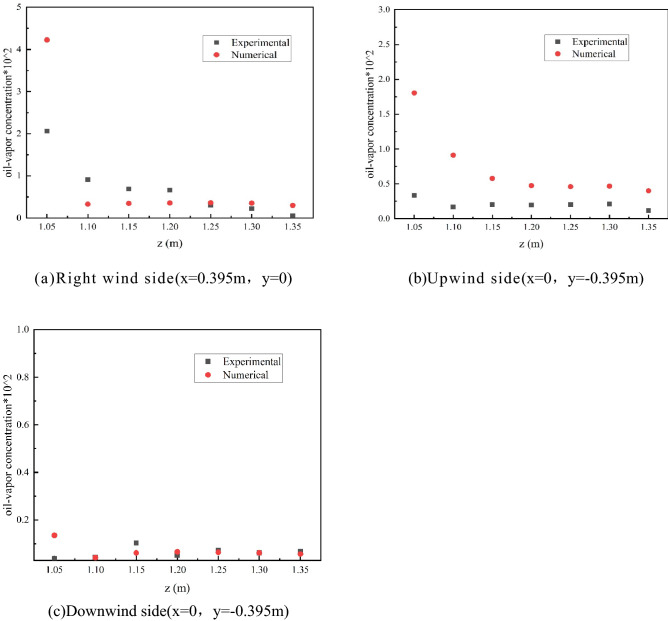
Experimental and simulated values of petroleum gas concentration in the space above the floating deck.

**Figure 3:**
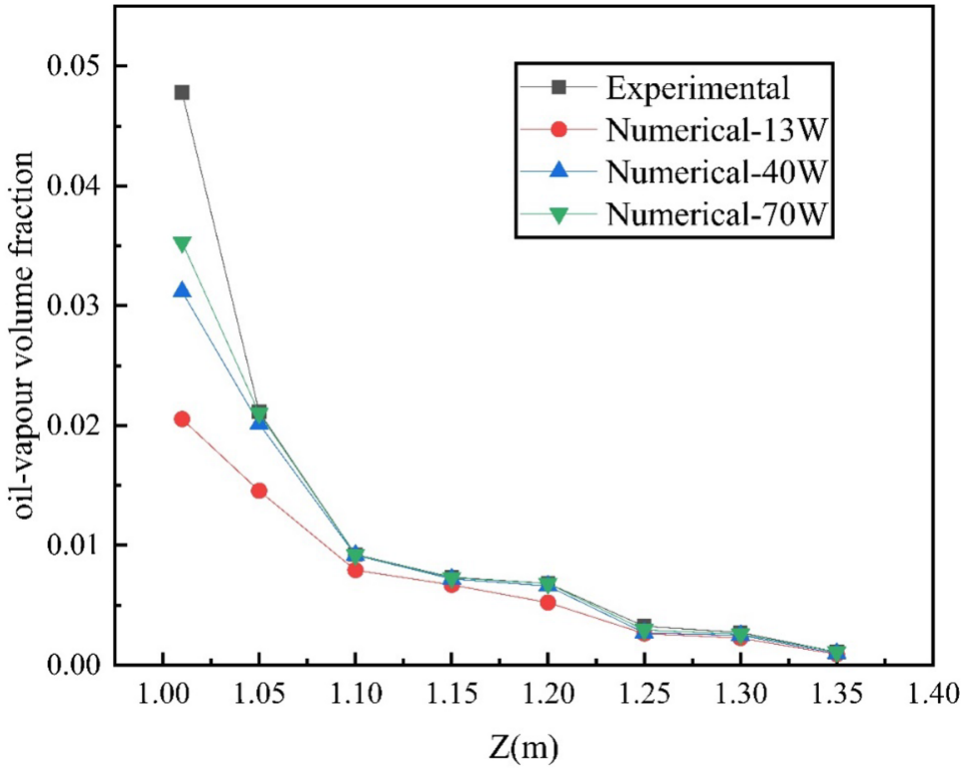
Grid independence verification.

In this paper, the concentration of petroleum gas is taken as the parameter of algorithm and grid independence verification. First, the parameter conditions and environmental scenes of petroleum gas leakage of small and medium-sized external floating roof tank sealing rings in the literature^14^ are selected for numerical simulation. Secondly, the experimental data of the concentration field above the floating deck are compared with the simulated values(as shown in Fig. 2). The concentration field is verified with the experimental data of external floating roof tanks in reference^10^. Finally, the numerical simulation is carried out by using grid models with the number of 0.13 + million, 0.40 + million, and 0.70 + million, respectively. As shown in Fig. 3.

It can be seen from the comparison between the experimental data and simulation data in Fig. 2 that the gas concentration value on the right side of the space above the floating plate is > windward side > downwind side, which indicates that the wind field distribution in the space above the floating deck is uneven, and the wind field in the downwind side of the space is disturbed greatly, and the gas concentrations are concentrated on both sides.When z ≥ 1.10, the simulated values of oil-vapor concentrations at lines A, B, and C are in good agreement with the actual values; and when z = 1.05 m, the monitoring point is close to the sealing ring, so the gas diffusion concentration will be on the high side. However, because the simulation condition is a simplification of the actual external environmental factors, the simulated value of this height is quite different from the experimental value, but the overall change trend is consistent. Therefore, it is reasonable to apply the simulation method and petroleum gas leakage model in this paper to the study of external floating roof tanks.

As can be seen from Fig. 3, the petroleum gas concentration value in the space above the floating deck in the simulation results is roughly consistent. Among them, the simulated value with 0.13 + million grids is quite different from the experimental value, which is larger than the simulated values with 0.40 + million grids and 0.70 + million grids, mainly because the grids in the storage tank area and leakage area are rough in the simulation with 0.13 + million grids. To ensure the accuracy of petroleum gas leakage diffusion concentration value in the floating deck leakage area of the tank group, this paper encrypts the leakage area of the storage tank. The results show that the experimental value and the simulated values of 0.40 + million and 0.70 + million grid sizes are pretty close. Still, there is a deviation between the initial concentration occurrence time of the two. Errors are mainly caused by test conditions, instrument accuracy, and other factors. Considering the computational performance and efficiency, this paper chooses a grid size of 0.40 + million.

### The example set and parameter definition

In this paper, we consider the concentration distribution of petroleum gas leakage from the sealing ring at different liquid levels and set up related calculations such as Table 2 below.

**Table 2:** Example settings (environmental parameters remain unchanged).

Environmental wind speed	Liquid level height					
	L = 6 m		L = 11 m		L = 16 m	
	Leakage rate (m/s)		Leakage rate (m/s)		Leakage rate (m/s)	
≥ 2.4 m/s	5	10	5	10	5	10
$\ge$ 5 m/s	5	10	5	10	5	10

In this paper, the space between the floating deck and the tank top is defined as the control area, the total height of the control area is h (unit: m) and the height above the floating deck from any z-direction section to the tank top is hi(i = 20%h ~ 80%h) (unit: m). Other specific parameters are defined in Table 3.

**Table 3:** Parameter definition.

Parameter definition	Symbol (unit)	Parameter definition	Symbol (unit)
Liquid level height	L (m)	Control area range (height of section from tank top)	h_i_(m)
Leakage volume	Q (m^3^/s)	Simulation of petroleum gas volume fraction	C
Average wind speed	V (m/s)	Fitting value of petroleum gas volume fraction	C’

## Results and discussion

### Petroleum gas diffusion flow field analysis

**Figure 4:**
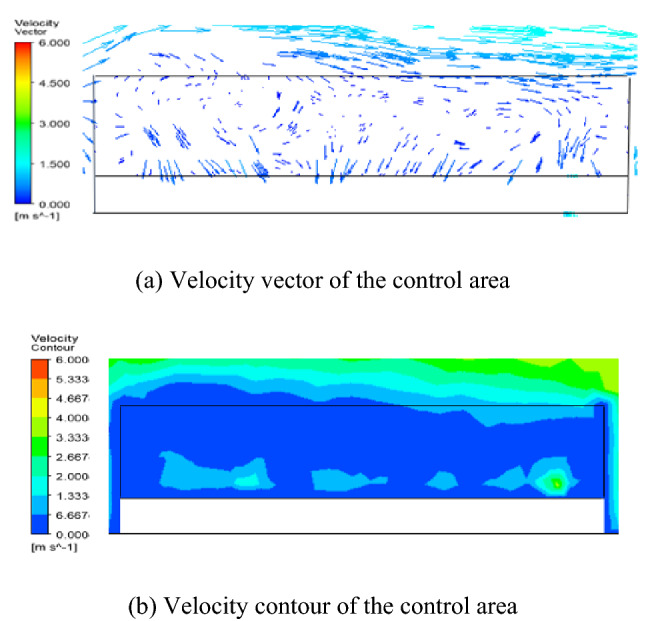
Simulation results of velocity field when L = 6 m.

**Figure 5:**
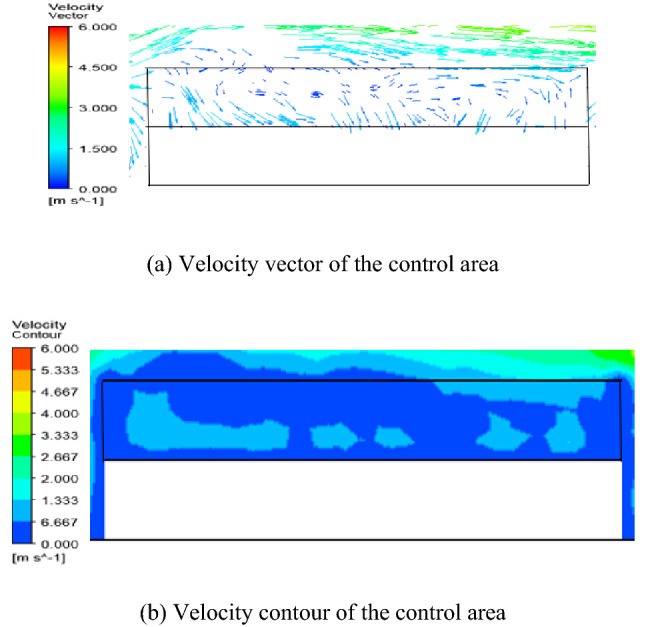
Simulation results of velocity field when L = 11 m.

**Figure 6:**
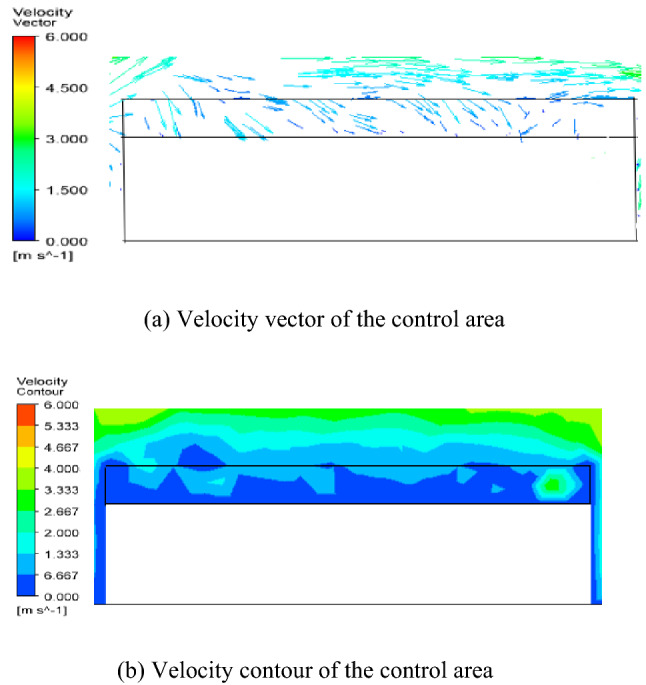
Simulation results of velocity field when L = 16 m.

The diffusion of petroleum gas after sealing ring leakage is mainly affected by airflow, so it is necessary to analyze the distribution of the airflow field. The flow field within the control area at different liquid levels is shown in Fig. 4, 5 and 6. The simulation results of the velocity field are similar to the literature^14^.

According to Figs. 4 and 5, it is known that due to the influence of wind speed, petroleum gas is blocked by the tank wall in the process of diffusion, which makes the airflow move in the opposite direction and form backflow. Under the interaction of jet and backflow, a vortex zone is formed in the control area. When L = 6 m, the liquid level is at the lower level of the tank, a large vortex is formed in the control area, the vortex volume in the center of the vortex is small, and a large wind speed area is formed near the tank wall, the wind speed distribution in the flow field is uneven, and the wind speed difference is the largest, as shown in Fig. 4; when L = 11 m, the liquid level is at the middle level of the tank, the vortex in the control area is reduced, the vorticity in the vortex center is small, the wind speed distribution in the flow field is uneven, and the wind speed difference is large, as shown in Fig. 5.

When L = 16 m, the liquid level is at a high level in the tank. Because the control area is close to the external flow field and affected by the horizontal transport of ambient wind, the overall distribution of wind speed in the flow field is uniform and the wind speed difference is small, as shown in Fig. 6.

According to Fig. 4, 5 and 6 and literature analysis, the results show that the flow field distribution in the control area is related to the liquid level height of the external floating roof tank. When the liquid level height is medium and low level, the airflow circulates in the space above the whole floating disc, the wind speed distribution in the control area is generally uneven, and the wind speed difference in the flow field is large. Airflow from the leeward into the space, floating plate above the floating plate to the upper lateral migration, from the upper side of the overflow, so the upper hand and leeward side of petroleum gas concentration are relatively low, the air above the floating disc space circular flow, and on both sides of tank position at the center of the vortex, petroleum gas concentration can accumulate over time lead to a higher concentration of petroleum gas on both sides of the position. when the liquid level height is a high level of the tank, the airflow only circulates in a small area near the tank wall on the upwind side, the wind speed distribution in the control area is uniform, and the wind speed difference in the flow field is small. The airflow enters the space above the floating disc from the upwind side and spills out to the downwind side along the floating disc, so the petroleum gas concentration distribution is more uniform, and the petroleum gas concentration value is lower than that of the petroleum gas leakage at the middle and low liquid level height.

**Figure 7:**
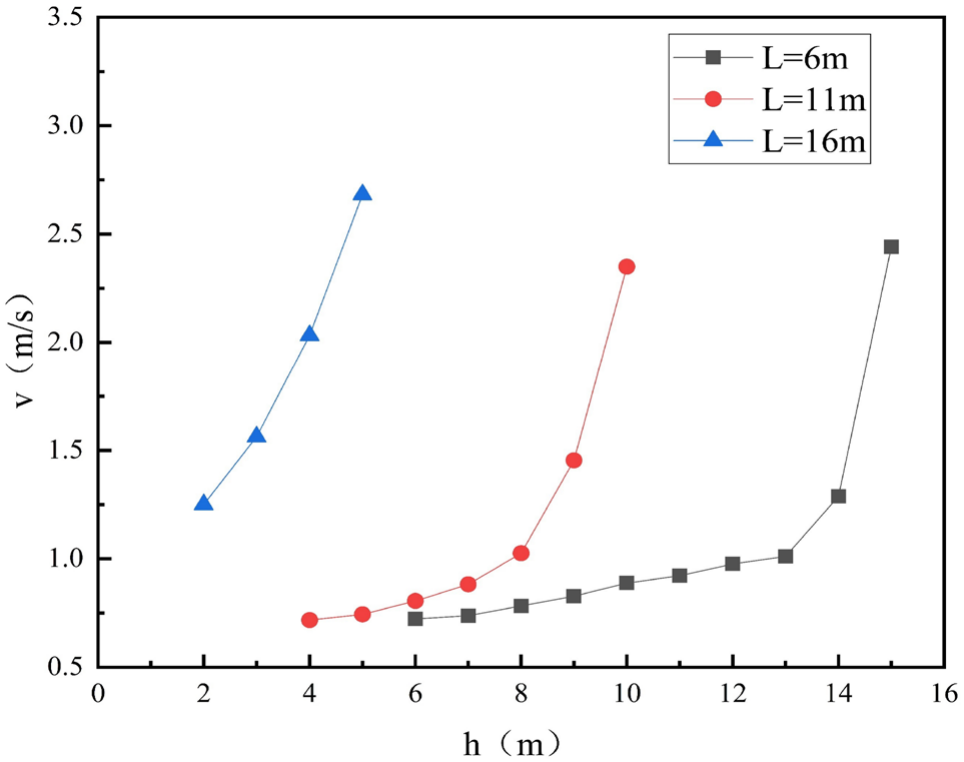
Variation of v with h at different within the control area.

Considering the inhomogeneity of the wind field in the control area, this paper selects the average wind speed of the cross-section for research and discusses the variation law of wind speed at different heights in the control area under different liquid levels, as shown in Fig. 7.

Figure 7 shows that with the increase of h, the wind speed in the control area at medium and low liquid levels is approximately exponentially distributed; when the liquid level is high, the change of wind speed in the control area is linearly related to h.

### Petroleum gas diffusion law in the space above floating roof under different liquid level heights

**Figure 8:**
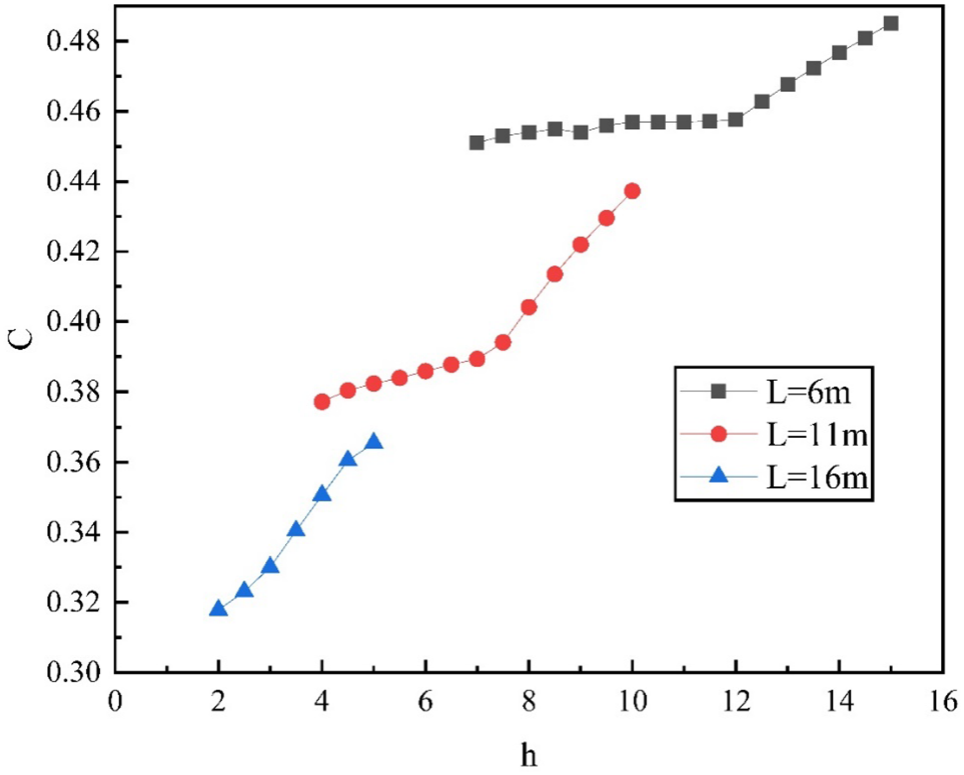
Variation of C with h in different liquid height control areas.

Set the liquid level height as the low, middle, and high liquid level of tank height (L). The law of petroleum gas diffusion in the liquid level of 30%L, 50%L, and 70%L while other states and parameters remain unchanged. According to the simulation results, the diffusion and stability stage of 600 s after ventilation is selected for analysis, and a section is taken every 0.5 m along the gas diffusion trajectory, and the variation of petroleum gas volume fraction in the space above the floating deck with distance when the liquid height: L1 = 6 m, L2 = 11 m, and L3 = 16 m are obtained as shown in Fig. 8.

As shown in Fig. 8, the C in the diffusion control area gradually increases from 0.45 and 0.38 to 0.46 and 0.39 with the increase of h for the liquid height is L1 = 6 m and L2 = 11 m; when hi ≥ 1/2 h, C increased significantly with the slope of 0.0075 and 0.013, respectively. When the liquid level height is L3 = 16 m, C in the control area increases linearly with a slope of 0.017. The reason is that, as shown in Figs. 4, 5, 6 and 7, the space above the middle and low liquid level is exposed to the ambient wind, and the interaction between jet and backflow forms a vortex area within the control area, so the C in this range shows a whirling upward trend. When the liquid level is high, the space above the floating deck is closest to the top outlet of the tank, and the wind field is highly disturbed, so the petroleum gas concentration increases linearly.

### Fitting and verification of petroleum gas distribution function

Considering that the change of v and h in the control area of the space above the floating roof tank at different times and different liquid heights are closely related to the distribution of petroleum gas concentration, Re dimensionless analysis is used to analyze the distribution law of petroleum gas flow field in the control area, and the specific formula is as follows:

(7) Re equation
  7 $$Re=\rho vL/\mu$$
  -ρ is Gas density (Take the average density of cross-section C_3_H_8_ in z-direction above the floating deck;)
  -v is the gas velocity (Take the average wind speed of the z-direction section above the floating deck;
  -L is characteristic length (Take the height of any section in the z-direction within the control area from the tank top and L is replaced by h_i_);
  -$\mu$ is a dynamic viscosity coefficient (Considering the main component of petroleum gas is propane (C_3_H_8_), it is selected as the petroleum gas parameter value; $\mu$: 6.75*${10}^{-6}$).

According to formula (3), it is known that the leakage volume (Q) has a significant influence on the gas diffusion concentration. Therefore, in this paper, when investigating the leakage of sealing ring at different levels from a floating tank, the relationship between C, Reynolds number (Re) during the diffusion of oil gases, and A_Q_ (the diffusion state of oil gases leaking into the control zone space is mainly considered). Among them:

$${A_{Q}}=Q* t/Q_{h}\,\,\,\, ({\text{t refers to time}; {Q_{h}} \text{is the volume within the control range}}).$$

### Fitting of petroleum gas distribution function

To directly calculate the petroleum gas volume concentration values under different conditions in the tank farm and obtain the functional relationship between C, Re, and A_Q_ after sealing ring leakage. Relevant calculation examples of sealing ring leakage at 30%L, 50%L, and 70%L liquid level height of storage tanks are set, as shown in Table 2.

**Figure 9:**
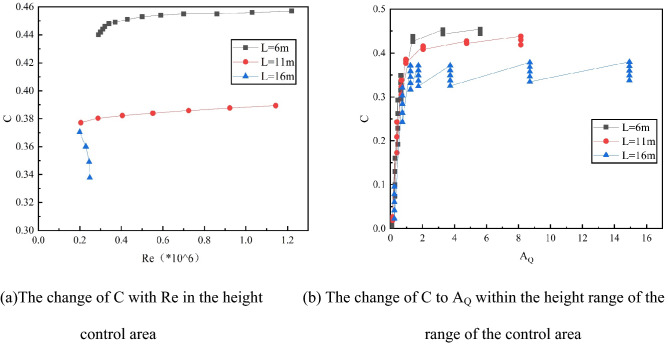
Variation of C with Re and A_Q_ in the height range of the control area.

First of all, according to the calculation results, we can get the Re at different liquid level heights. Second, considering the relationship between Re with A_Q_ and C within the control area, we can draw the relationship diagram between the change of C and Re, and A_Q_ within different control area heights, as shown in Fig. 9.

According to Fig. 9, it is concluded that the C in the control area is directly proportional to the Re and A_Q_ of the space height above the floating deck when the petroleum gas leakage of the middle and low liquid level sealing ring occurs, and inversely proportional to the Re and A_Q_ of the high liquid level height. The C in the control area is inversely proportional to the liquid level height L. The fitting process of the C distribution function in the control area is shown in Table 4.

**Table 4:** Concentration fitting steps at different liquid level heights.

Liquid height	The fitting function	R^2^
L = 6 m	$C=0.475-0.0098Re-0.0087/Re$ $C=0.505-0.18/{A}_{Q}+0.041/{A}_{Q}^{1.5}$	0.9774
L = 11 m	$C=0.383+0.0063Re-0.0015/Re$ $C=0.46-0.13/{A}_{Q}+0.027/{A}_{Q}^{1.5}$	0.99
L = 16 m	$C=2.002-3.983Re-0.167/Re$ $C=0.362-0.053/{A}_{Q}^{1.5}+0.007/{A}_{Q}^{2}$	0.97
Summary (Re): C = ${P}_{1}Re+{P}_{2}/Re+{P}_{3}$; Summary (Q): Let ${A}_{Q}^{^{\prime}}=1/{A}_{Q}$, then: C = ${P}_{1}{A}_{Q}^{^{\prime}}+{P}_{2}{{(A}_{Q}^{^{\prime}})}^{1.5}+{P}_{3}$, ($30\%L\le$ L $\le 50\%L$) C = ${P}_{1}{{(A}_{Q}^{^{\prime}})}^{1.5}+{P}_{2}{{(A}_{Q}^{^{\prime}})}^{2}+{P}_{3}$, (50 $\%<L\le 70\%L$) (P_1_, P_2_, and P_3_ are constant.)		

From Table 4, it can be concluded that when 30%L ≤ L ≤ 70%L, the C of the diffusion region has a change in approximate power function with the Re and exists a nonlinear increasing trend with A_Q_ as a whole: When the A_Q_ increases, the C in the early control area gradually rises with the increase of the liquid level height; when the leakage is constant, the C in the control area gradually decreases with the rise of liquid level height. The main reasons are that the height of the control area is close to the tank top so the concentration of petroleum gas is affected by fresh airflow, and the concentration is low. The R^2^ values of function fitting within the height range of the control area are all greater than 0.97. Therefore, in this paper, the curve fitting method is used to study the variation law of petroleum gas concentration when sealing ring leaks at a 30–70% liquid level.

Secondly, according to the concentration function shown in Figs. 8, 9, and above, the distribution function of sealing ring gas concentration with high, medium, and low liquid levels is different in the control area when petroleum gas leaks. Therefore, the simulation data of petroleum gas leakage from sealing rings with different liquid levels are deduced and fitted. It is concluded that when gas leakage from sealing rings with 30% L to 70% L liquid levels occurs, the functional relationship between C, Re, and A_Q_ in the height of the diffusion zone is as follows:

(8) When 30%L sealing ring leaks petroleum gas:
  8 $$\mathrm{C}=0.015Re-0.132{A}_{Q}^{^{\prime}}+0.008{({A}_{Q}^{^{\prime}})}^{2}+0.4869$$

(9) When 50%L sealing ring leaks petroleum gas
  9-1 $$\mathrm{C}=0.036Re-0.144{A}_{Q}^{\mathrm{^{\prime}}}+0.01{({A}_{Q}^{\mathrm{^{\prime}}})}^{2}+0.495$$
  Considering the leakage of 30%L and 50%L sealing ring, the change regularity of C in the control area is strongly related and the basic relationship of the function is approximate, so the concentration distribution function of 30%L ≤ L ≤ 50%L is fitted:
  9-2 $$\mathrm{C}=0.026Re-0.138{A}_{Q}^{^{\prime}}+0.009{({A}_{Q}^{^{\prime}})}^{2}+0.491$$

(10) When 70%L sealing ring leaks petroleum gas
  10 $$\mathrm{C}=0.21Re-0.039{A}_{Q}^{^{\prime}}-0.011{({A}_{Q}^{^{\prime}})}^{2}+0.316$$
  where A_Q_ is the leakage state within the control area after gas leakage; Re is the Reynolds number; C is the volume fraction of petroleum gas.

To sum up, when the wind speed is 2.4 m/s, and the height of the control area is 20%-80% of the space above the floating deck when the liquid level is leaking at high, medium, and low levels, the distribution function of petroleum gas concentration above the storage tank is as follows (11)–(12):

(11) When $$30\boldsymbol{\%}\le \mathrm{L}\le 50\boldsymbol{\%}$$
  11 $$\mathrm{C}=0.026Re-0.138{A}_{Q}^{^{\prime}}+0.009{({A}_{Q}^{^{\prime}})}^{2}+0.491$$
(12) When $$50\boldsymbol{\%}<\mathrm{L}\le 70\boldsymbol{\%}$$
  12 $$\mathrm{C}=0.21Re-0.039{A}_{Q}^{^{\prime}}-0.011{({A}_{Q}^{^{\prime}})}^{2}+0.316$$

Combined with the prediction of gas diffusion concentration, the reasonable rescue time can be known. As shown in Table 5.

**Table 5:** Under different liquid levels, the leakage of gas corresponds to the required emergency rescue time.

Liquid level height	Total Leakage(m^3^/s)	Emergency rescue time
30% ≤ L ≤ 50%	> 750	≤ 20 s
	375 < Q ≤ 750	20 s ≤ t ≤ 40 s
50% ≤ L ≤ 70%	> 750	≤ 30 s
	375 < Q ≤ 750	30 s ≤ t ≤ 1 min

When the petroleum gas concentration within the control area in the tank does not reach 0.26, it means that the petroleum gas concentration in the whole tank still meets the safety standard. When the leakage of a particular liquid level in the tank and the leakage area is known, according to the corresponding formulas of 30% ≤ L ≤ 50% and 50% < L ≤ 70%, it can be calculated whether the petroleum gas concentration value C in the control area of the tank reaches the petroleum gas explosion risk value.

By fitting the functional relationship of L, A_Q_, Re, and C, the time required for the petroleum gas diffusion concentration to reach a safe value within the control area after leakage can be calculated under certain conditions.

### Verify the accuracy of the distribution function

**Figure 10:**
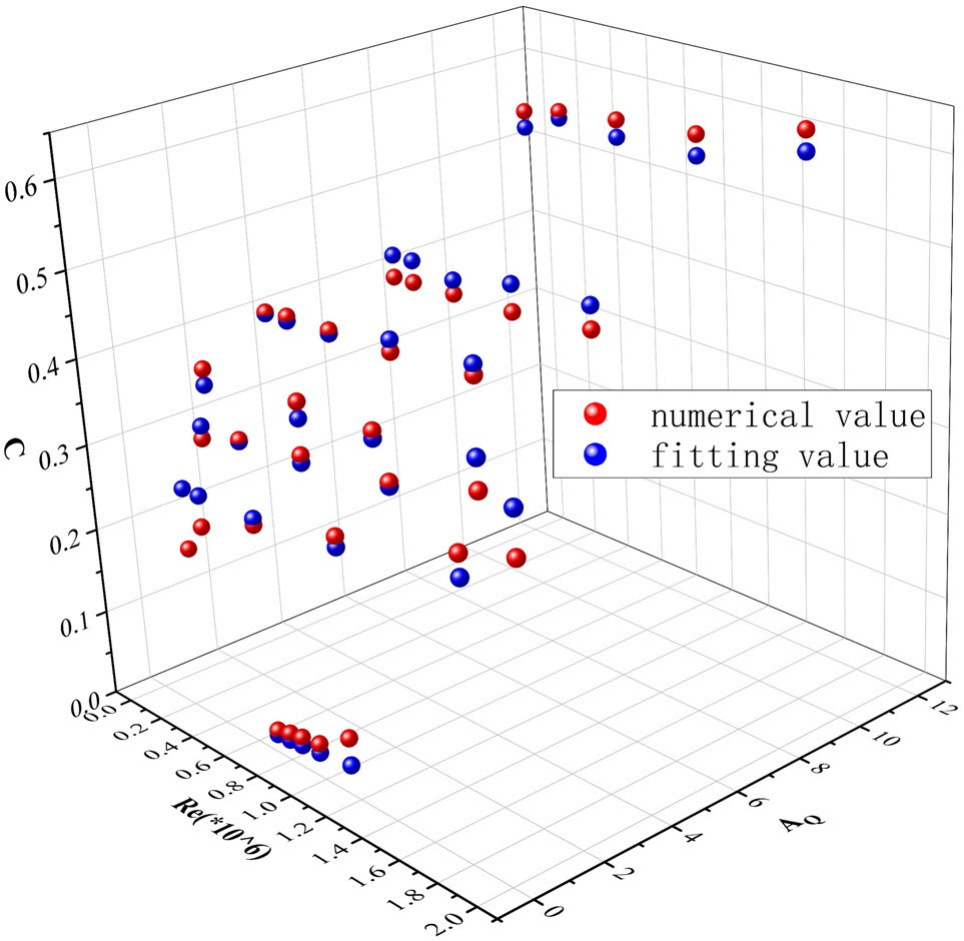
Comparison of simulated and fitted values when L = 14 m.

Compared to the numerical simulation value and the fitting value when L = 14 m, as shown in Fig. 10. The red dots in the figure represent the simulated values in the control area, and the blue dots represent the fitted values of the formula. The x-axis is Re, the y-axis is A_Q_, and the z-axis is C (gas volume fraction).

When the liquid height L = 14 m is selected for the petroleum gas leakage of the sealing ring, the wind speed is 5 m/s and the mass velocity is 5 m/s, which is used to verify the petroleum gas diffusion concentration of the sealing ring leaking at the medium and low liquid level. When L = 14 m, the error between the simulation and the fitting calculation of petroleum gas concentration is shown in Fig. 10. It can be concluded that the variation law of petroleum gas concentration with liquid level height is approximated by numerical simulation and formula calculation, and the absolute error range between the simulated value and the calculated value is 7%.

These errors are primarily attributable to the following factors: (1) The petroleum gas leakage of the sealing ring is mainly volatile organic compounds. Considering that the main component of gas is propane and the physical properties of the gas are close to petroleum gas so that propane is used to replace the petroleum gas after the leakage of the sealing ring in the simulation. (2) Only eight measuring points are set for each section in the simulation. However, the calculated value of the formula is the average concentration of the cross-section, and there will be errors if the measurement points are few. (3) Numerical simulation is carried out based on several assumptions and the calculation results are ideal. In addition, simulation factors such as mesh quality, turbulence model selection, etc. will also bring errors that cannot be ignored. Generally speaking, in the case of the same leakage amount, the RSD is less than 3% within the control area of different liquid level heights.

In the existing storage tank base, the excessively high diffusion concentration of petroleum gas leakage from the sealing ring of the standard large-scale external floating roof tank will seriously affect the safe operation of the tank farm and the occupational health of workers. However, only three basic parameters (leakage height, leakage, Reynolds number) can be substituted into the proper function to calculate the petroleum gas volume fraction at a height from a monitoring point above the floating deck. The process fitted according to the simulation results is suitable for the petroleum gas diffusion law of similar storage tanks.

## Conclusion

In this paper, the variation law of gas leakage diffusion concentration of sealing rings with different liquid levels of external floating roof tanks is studied with Fluent software. The diffusion characteristics of petroleum gas under various conditions are simulated and the distribution law of gas concentration is obtained. The main conclusions are as follows:

1. When the wind speed and leakage volume are constant, the change of gas concentration in the height of the control area is inversely proportional to the liquid level height under the external floating roof tank. H is greater, that is, the greater the distance between the monitoring points in the control area and the top of the tank, the higher C is.
2. There is a specific correlation between the C in the control area and Re. The diffusion concentration of gas leakage of the middle and low liquid level sealing ring is proportional to the Re at first and then gradually stabilizes. When the high-level sealing ring leaks, the wind field above the floating deck is highly disturbed and the gas diffusion concentration whole decreases. In general, the C is the form of a power function with the increase of Re.
3. The Q will affect the diffusion concentration of petroleum gas after the sealing ring leaks at different liquid level heights. The C within the control zone increases with A_Q_. Likewise, when the Q is constant, the C in the control area is affected by the fresh airflow leading to the C gradually decreasing with the increase of the liquid level. In particular, the C is the greater change under the high liquid level. Therefore, the distribution of gas concentration can be regarded as two diffusion processes of medium and low liquid level and high liquid level.
4. Different conditions have different effects on the diffusion of petroleum gas. By analyzing a large number of simulated data, the functional relationship between C and Re, A_Q_, and L is determined after the sealing ring. The accuracy of the distribution function is verified by comparison with the numerical simulation data. This distribution function is suitable for the same type of petroleum gas leakage in storage tanks. It can predict the approximate range of the concentration of petroleum gas diffusion in the space above the floating deck and determine the corresponding emergency rescue time according to the concentration range, which has essential reference significance.

The research results are helpful for the rescuers to reasonably arrange the emergency rescue time when the petroleum gas leakage of the external floating roof tank does not reach the explosion risk within the control area. In the follow-up, the related research on the prediction of oil and gas leakage diffusion concentration under the condition of ignoring the size of the storage tank is mainly carried out.

## Supplementary Information

Supplementary Information 1.

Supplementary Information 2.

## Data Availability

The datasets used and/or analyzed during the current study are available from the corresponding author upon reasonable request. All data generated or analyzed during this study are included in this published article [and its supplementary information files].
