# Supplementary material for: Forecasts for the concentration of petroleum gas leakage diffusion under different liquid level heights of a sealing ring of sizeable floating roof tank
Source: Sci Rep. 2022 Nov 9;12:19124. doi: 10.1038/s41598-022-22934-2 (PMC9646790; doi:10.1038/s41598-022-22934-2)
Supplement: Supplementary file 1 — Supplementary Information 1. [file 41598_2022_22934_MOESM1_ESM.docx]

**1.Manuscript title：**

**Forecasts for the concentration of petroleum gas leakage diffusion under different liquid level heights of a sealing ring of sizeable floating roof tank**

**2.Author list：**

**Yonghui Wei ^1, a^, Wenfeng Wu ^1,2,*^, Hongliang Yu ^3,*^, Jinshu Lu ^1,∗^, Mengqing Huang ^1,+^ and Min Guo ^1,+^**

**^1^Zhejiang Ocean University, Department of Naval Architecture and Maritime, Zhoushan**, **316022, China**

**^2^Brunel University, Department of Mechanical and Aerospace Engineering, London, England**

**^3^Yantai University, School of the ocean, Yantai,264005, China**

First author: Yonghui Wei, Zhejiang Ocean University, Zhoushan,316022，Zhejiang, China. email: yh_wei1900266206@163.com, phone number:15255550046. ORCID: 0000-0001-9841-6685

First correspondent: Wenfeng Wu, Zhejiang Ocean University, Zhoushan,316022，Zhejiang, China.& Department of Mechanical and Aerospace Engineering, Brunel University, London, England; email: wuwenfeng0611@126.com, phone number:13868238146. ORCID: 0000-0002-1349-2820

Second correspondent: Hongliang Yu. School of ocean Yantai University, Shandong, China. email: yuhongliang@ytu.edu.cn, phone number:15998497425. ORCID: 0000-0002-7245-2538

Third correspondent: Jinshu Lu. Zhejiang Ocean University, Zhoushan,316022，Zhejiang, China. email: ljs_ljs@zjou.edu.cn. ORCID: 0000-0003-1421-5478

Mengqing Huang .Zhejiang Ocean University, Zhoushan,316022，Zhejiang, China. email: huangmengqing1998@163.com. ORCID: 0000-0002-7815-1509

Min Guo. Zhejiang Ocean University, Zhoushan,316022，Zhejiang, China. email: GUOMIN@zjou.edu.cn. ORCID: 0000-0002-8355-4628

Table 1. The setting of boundary conditions and initial conditions

| Boundary conditions | Setting boundaries | Initial condition | Parameter setting |
| --- | --- | --- | --- |
| Calculation domain left entrance（right exit） | Velocity-inlet  （Pressure-outlet） | temperature | 300K |
| Calculate the top and both sides of the domain boundary | Symmetry | atmospheric pressure | 101325Pa |
|  |  | atmospheric stability | D(-1°C/m) |
| Tank wall and ground | Wall | Mass flow of leakage port | 10m/s |
| Tank top | Interior | wind speed（satisfy the formula (Chi et al. 2007) ） | $u_{z}=u_{1}\frac{lnz-lnz_{0}}{lnz_{1}-lnz_{0}}$ |
| sealing ring | Velocity-inlet |  |  |
| Model and algorithm: k-ε model, Simple standard algorithm | | | |

Table 2. Example Settings (environmental parameters remain unchanged)

| Liquid level height  Environmental  wind speed | L=6m | | L=11m | | L=16m | |
| --- | --- | --- | --- | --- | --- | --- |
| $\geq$2.4m/s | Leakage rate （m/s） | | Leakage rate （m/s） | | Leakage rate （m/s） | |
|  | 5 | 10 | 5 | 10 | 5 | 10 |
| $\geq$5m/s | 5 | 10 | 5 | 10 | 5 | 10 |

Table 3. Parameter definition

| parameter definition | Symbol (unit) | parameter definition | Symbol (unit) |
| --- | --- | --- | --- |
| Liquid level height | L（m） | Control area range (height of section from tank top) | h_i_(m) |
| Leakage volume | Q（m^3/s） | Simulation of petroleum gas volume fraction | C |
| Average wind speed | v（m/s） | Fitting value of petroleum gas volume fraction | C’ |

Table 4. Concentration fitting steps at different liquid level heights

| Liquid height | The fitting function | R^2^ |
| --- | --- | --- |
| L=6m | $C=0.475-0.0098Re-{0.0087}/{Re}$  $C=0.505-{0.18}/{A_{Q}}+{0.041}/{A_{Q}^{1.5}}$ | 0.9774 |
| L=11m | $C=0.383+0.0063Re-{0.0015}/{Re}$  $C=0.46-{0.13}/{A_{Q}}+{0.027}/{A_{Q}^{1.5}}$ | 0.99 |
| L=16m | $C=2.002-3.983Re-{0.167}/{Re}$  $C=0.362-0.053/A_{Q}^{1.5}+{0.007}/{A_{Q}^{2}}$ | 0.97 |
| Summary（Re）： C=$P_{1}Re+{P_{2}}/{Re}+P_{3}$;  Summary（Q）: Let$A_{Q}^{'}=1/{A_{Q}}$, then:  C=$P_{1}A_{Q}^{'}+P_{2}{{(A}_{Q}^{'})}^{1.5}+P_{3}$ ,（$30\%L\leq$L$\leq50\%L$）  C=$P_{1}{{(A}_{Q}^{'})}^{1.5}+P_{2}{{(A}_{Q}^{'})}^{2}+P_{3}$,（50$\%<L\leq70\%L$）  (P_1_, P_2_, and P_3_ are constant.) | | |

Table 5. Under different liquid levels, the leakage of gas corresponds to the required emergency rescue time

| Liquid level height | Total Leakage(m^3^/s) | Emergency rescue time |
| --- | --- | --- |
| 30％≤L≤50％ | ＞750 | ≤20s |
|  | 375＜Q≤750 | 20s≤t≤40s |
| 50％≤L≤70％ | ＞750 | ≤30s |
|  | 375＜Q≤750 | 30s≤t≤1min |
